# Transgenerational plasticity of inducible defences: Combined effects of grand‐parental, parental and current environments

**DOI:** 10.1002/ece3.6046

**Published:** 2020-02-14

**Authors:** Juliette Tariel, Sandrine Plénet, Émilien Luquet

**Affiliations:** ^1^ Univ Lyon, Université Claude Bernard Lyon 1, CNRS, ENTPE, UMR 5023 LEHNA Villeurbanne France

**Keywords:** carry‐over effect, multigenerational effect, phenotypic plasticity, *Physa acuta*, predator‐prey interactions

## Abstract

Phenotypic plasticity can occur across generations (transgenerational plasticity) when environments experienced by the previous generations influenced offspring phenotype. The evolutionary importance of transgenerational plasticity, especially regarding within‐generational plasticity, is a currently hot topic in the plasticity framework. How long an environmental effect can persist across generations and whether multigenerational effects are cumulative are primordial—for the evolutionary significance of transgenerational plasticity—but still unresolved questions. In this study, we investigated how the grand‐parental, parental and offspring exposures to predation cues shape the predator‐induced defences of offspring in the *Physa acuta* snail. We expected that the offspring phenotypes result from a three‐way interaction among grand‐parental, parental and offspring environments. We exposed three generations of snails without and with predator cues according to a full factorial design and measured offspring inducible defences. We found that both grand‐parental and parental exposures to predator cues impacted offspring antipredator defences, but their effects were not cumulative and depended on the defences considered. We also highlighted that the grand‐parental environment did alter reaction norms of offspring shell thickness, demonstrating an interaction between the grand‐parental transgenerational plasticity and the within‐generational plasticity. We concluded that the effects of multigenerational exposure to predator cues resulted on complex offspring phenotypic patterns which are difficult to relate to adaptive antipredator advantages.

## Graphical abstract

How long an environmental effect can persist across generations and whether multigenerational effects are cumulative are primordial—for the evolutionary significance of transgenerational plasticity—but still unresolved questions. We exposed three generations of *Physa acuta* snails without and with predator cues according to a full factorial design and measured offspring inducible defences. We found that both grand‐parental and parental exposures to predator cues impacted offspring antipredator defences, but their effects were not cumulative and depended on the defences considered.

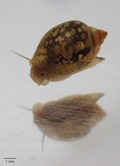

## 1 INTRODUCTION

Organisms may respond to fluctuating environments by adapting through genetic evolution over generations or through phenotypic plasticity. This last is traditionally defined as the capacity of a given genotype to produce alternative phenotypes under different environmental conditions (within‐generational plasticity) (Pigliucci, 2005; West‐Eberhard, 2003). Plasticity may also occur across generations (transgenerational plasticity), when the phenotype of offspring is influenced by carry‐over effects of past environments experienced by the previous generation(s) (Agrawal, Laforsch, & Tollrian, 1999; Galloway & Etterson, 2007; Salinas, Brown, Mangel, & Munch, 2013). Ancestors can alter the phenotype of their offspring without involving changes in nucleotide sequence through a range of nongenetic processes as parental effects, *for example,* transmission of nutrients, hormones, proteins (Crean & Bonduriansky, 2014; Mousseau & Fox, 1998), or by any form of epigenetic inheritance, *for example,* DNA methylation marks, histone protein modifications, noncoding small RNAs (Holeski, Jander, & Agrawal, 2012; Schlichting & Wund, 2014). Transgenerational plasticity has been shown for several animal and plant taxa, various traits (behavior, morphology, and life‐history) in response to abiotic (*e.g.,* temperature, salinity, contaminants) and biotic (*e.g.,* predation) environments (Bonduriansky & Day, 2009; Donelson, Salinas, Munday, & Shama, 2018; Salinas et al., 2013). Transgenerational plasticity may enable organisms to cope with fast‐changing environments because it refines offspring phenotype in anticipation of the environmental conditions they are likely to experience (Bonduriansky & Day, 2009; Donelson et al., 2018; Herman & Sultan, 2011).

However, while the main effects of parental environment and even grand‐parental one on offspring phenotype are now widely observed (*e.g.,* Mousseau & Fox, 1998; Wolf & Wade, 2009; Herman & Sultan, 2011; Donelson et al., 2018), we need to investigate how the combined effects of multigenerational environments shaped the offspring phenotype to properly assess the potential for adaptive transgenerational effects (Prizak, Ezard, & Hoyle, 2014). Such investigations require to perform factorial experiments that often lead to complex patterns of phenotypic offspring responses. For example, Hafer, Ebil, Uller, and Pike (2011) and Walsh, Whittington, and Funkhouser (2014) in collembolan (*Folsomia candida*) and cladoceran (*Daphnia ambigua*), respectively, demonstrated that age and length at maturity were affected by an interactive effect between grand‐parental and parental environments. Moreover, such combinations of grand‐parental and parental effects can also depend on the offspring environmental context. Plaistow, Lapsley, and Benton (2006), for instance, showed in the soil mite *Sancassania berlesei* that the persistence of past environments (across four generations) differed between high‐ and low‐food offspring contexts. In this study, our aim was to investigate how the grand‐parental, parental and offspring exposures to predator cues combine to shape the predator‐induced defences of offspring.

**Figure 1:**
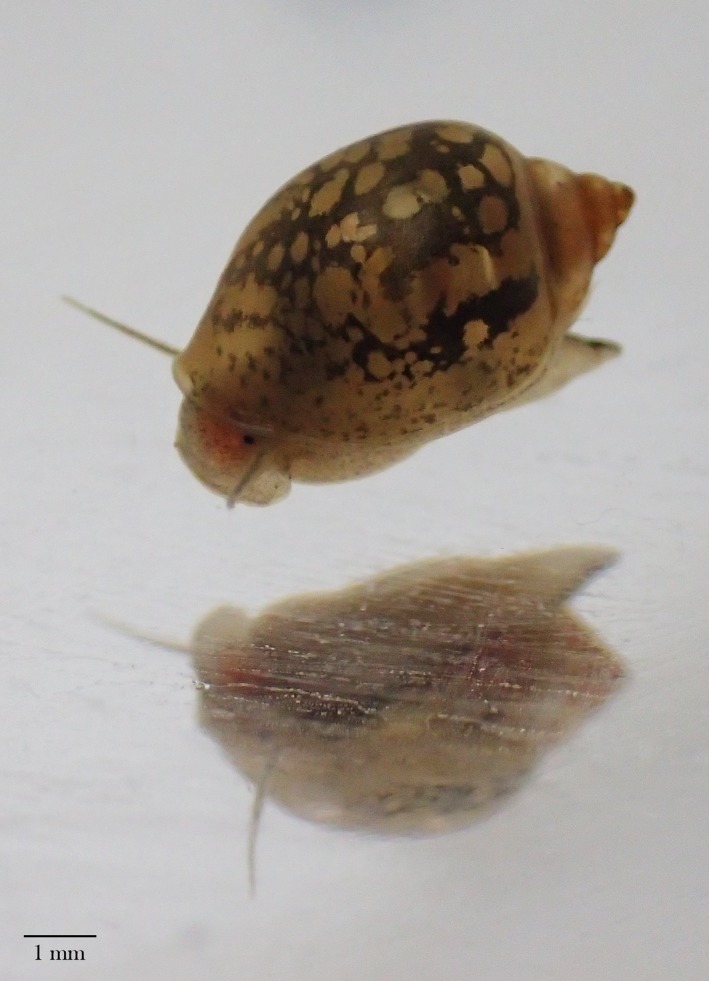
*Physa acuta* is a freshwater and simultaneous hermaphroditic snail, invasive from North America (Lydeard, Campbell, & Golz, 2016). This adult *P. acuta* is crawling underwater and is reflected on the bottom of the rearing vial

Predator‐induced plasticity is a well‐known model in within‐generational plasticity study (*e.g.,* Harvell, 1990; Relyea, 2001; Hoverman, Auld, & Relyea, 2005) and allows an individual to fine‐tune its phenotypes facing predation risk (Benard, 2004; Lima, 1998; Tollrian & Harvell, 1999). Predator‐induced defences are also widely used to investigate transgenerational plasticity over two generations (parental and offspring generations) (*e.g.,* Agrawal et al., 1999; Walsh, Cooley, Biles, & Munch, 2015; Bell & Stein, 2017; Colicchio, 2017; Sentis et al., 2018). We focused on a hermaphroditic gastropod *Physa acuta* (Figure 1). Physidae are well‐known to develop adaptive phenotypes in response to predation risk (Auld & Houser, 2015; Auld & Relyea, 2008, 2011; Beaty et al., 2016; DeWitt, Sih, & Hucko, 1999; Gustafson, Kensinger, Bolek, & Luttbeg, 2014). Predator (crayfish) cues induce within‐generational plasticity of *Physa sp.* life‐history traits (delay of age at maturity but at a larger size; Auld & Relyea, 2008), shell thickness (thicker shell; Auld & Relyea, 2011), shell size (narrower shape; DeWitt, 1998), and escape behavior (crawling‐out the water; Alexander & Covich, 1991; DeWitt et al., 1999).

According to a full factorial design, three successive generations of snails from hatching to sexual maturity were exposed or not to predator cues. The results concerning the first two generations have demonstrated a predator‐induced transgenerational plasticity in *P. acuta* (Luquet & Tariel, 2016) that has been confirmed in a concomitant study (Beaty et al., 2016). Here, we focused on the F3 generation to investigate how the effects of grand‐parental, parental and offspring environments combine to influence escape behavior, shell thickness, and shell morphology. First, we expected that both grand‐parental and parental exposures to predator cues influence the offspring phenotypes. In addition, as we already observed that parental environment can interact with the offspring environment (Luquet & Tariel, 2016), we expected that the offspring phenotypes result from a three‐way interaction among grand‐parental, parental and offspring environments.

## 2 METHODS

### 2.1 Animal collection and experimental design

**Figure 2:**
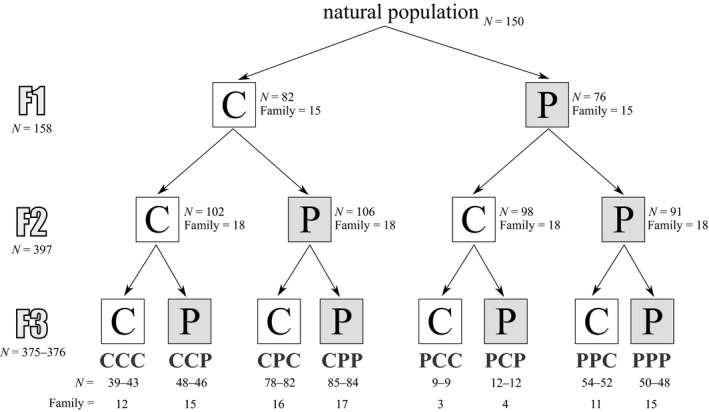
Number of individuals (*N*) and families (Family) at each generation (F1, F2, and F3). "C" stands for control environment and "P" for predator‐cue environment. For the F3 generation, two number of individuals are reported, one for behavioral measurements (first position) and one for other measurements (second position)

Adult *P. acuta* snails (Figure 1) were collected on March 2015 in a lentic backwater of the Rhône river (45° 48'6"N, 4° 55'33"E) in Lyon, France. The wild‐caught adult snails constituted our F0 generation (see Appendix 1 for a figure of the experimental design). We pooled them overnight in a 10L vial to ensure that offspring result from outcrossing (*P. acuta* is a preferential outcrosser; Jarne, Pointier, David, & Koene, 2010). Then, we individually isolated all F0 snails in 70 ml plastic vials filled with reconstituted water (2.4 g NaHCO3, 3 g CaSO4, 1.5 g MgSO4, 0.1 g KCl to 25 L deionized water) in a 25°C experimental room with 12 hr light‐dark photoperiod. After 24 hr, we removed the F0 adults from the vials and we randomly choose 15 vials with one egg capsule each. These 15 egg capsules constituted our 15 maternal families (hereafter called only “families”) of the F1 generation and developed until hatching (~7 days). Two days after hatching, we randomly sampled 12 siblings per family and split them into two environments: 6 snails remained in a no‐predator environment (control environment) while 6 others were moved in a predator‐cue environment. These F1 snails were reared in 70 ml plastic vials with their siblings until 28 days old where they were isolated in the same type of plastic vials until 35 days old. In order to generate the F2 generation, we made 6 F1 mating‐groups per treatment. A mating‐group was composed of 15 F1 snails (one F1 snail from each of the 15 families) in a 5L vial. We let the F1 snails to mate for 24h and then isolated them in a no‐predator water to ensure embryos were not exposed to predator environment. We randomly subsampled 18 F1 snails that had laid eggs from each treatment to generate the F2 generation. We then followed the same protocol as previously to rear F2 snails in control and predator‐cue environments according to a full factorial design until 49 days old. The F3 generation was then generated and reared using the procedure described above. As growth rate was slowing down every generation under our laboratory conditions, we let F3 snails grew up to a later age (74 days old) to ensure a sufficient size for measurements. This F3 generation was represented by eight combinations of grand‐parental (E1), parental (E2), and offspring (E3) environments: CCC, CPC, PCC, PPC, CCP, CPP, PCP, and PPP with each time “C” for control environment and “P” for predator‐cue environment (Figure 2). The number of individuals and families per combination of environments is reported on Figure 2.

Water and food (*ad libitum*, chopped and boiled lettuce) were renewed for all experimental snails twice a week. Predator‐conditioned water with predator cues was obtained by individually rearing crayfishes (*Procambarus clarkii*) in 4L reconstituted water and feeding with one crushed *P. acuta* adult one day before water change (Auld & Relyea, 2011). This crayfish‐conditioned water was used for the predator‐cue treatment while only reconstituted water was used for the control treatment. This crayfish species coexists with *P. acuta* in its native location in North America.

### 2.2 Measuring phenotypes

We measured escape behavior, shell thickness, snail mass, and four shell size traits on F3 (offspring) snails. We assessed escape behavior three times in 70 days old F3 snails through three consecutive days starting one day after the water change. We recorded the position above/on or below the water surface in the rearing vials with predator cues present or absent according to the treatment. Crawling‐out of the water (position above water surface) is considered as allowing to escape from benthic predators like crayfishes (DeWitt et al., 1999).

At 74 days old, we gently dried snails with paper towel and measured the snail total wet mass (body and shell) with an electronic scale at the nearest 0.001 mg. A photograph of each snail aperture upwards was taken with an Olympus SC50 camera installed on an Olympus SZX9 binocular and its Olympus DF PLAPO 1X‐2 objective at a ×8 magnification. Shell and aperture length and width were measured on these photographs with the software ImageJ (Schneider, Rasband, & Eliceiri, 2012). Shell thickness was measured with an electronic calliper at the nearest 0.01 mm at the edge of the aperture. Shorter and narrower shell and aperture dimensions (after adjusting for mass) and thicker shell are adaptive antipredator responses (Auld & Relyea, 2011).

### 2.3 Statistical analysis

The multigenerational effects of predator cues on escape behavior (*i.e.*, snail position above/on or below the water surface) were analyzed using generalized linear mixed models (GLMM) assuming a binomial distribution (logit link function). Grand‐parental (E1), parental (E2), offspring (E3) environments, and all interactions were considered as fixed effects. Family and individual identity (to account for repeated measures on the same individual) were considered as random effects. We tested significance of fixed and random effects with likelihood ratio tests.

To analyze the multigenerational effects of predator cues on snail mass, shell thickness, shell length, shell width, aperture length, and aperture width, we performed a principal component analysis in order to extract the first and second principal components (PC1 and PC2), both explaining 96% of the variance.

We then used PC1 and PC2 as response variables in two linear mixed models where grand‐parental (E1), parental (E2), offspring (E3) environments, and all interactions were considered as fixed effects. Family was considered as a random intercept. We used restricted maximum likelihood estimation and Kenward and Roger's approximation for degrees of freedom. We tested significance of fixed effects with type II *F*‐tests (Kuznetsova, Brockhoff, & Christensen, 2017) and significance of random effect with likelihood ratio test.

All statistical analyses were performed with R 3.4.1 (R Core Team, 2017) and with the packages lme4 (Bates, Mächler, Bolker, & Walker, 2015) and FactomineR (Lê, Josse, & Husson, 2008).

## 3 RESULTS

### 3.1 Escape behavior

**Figure 3:**
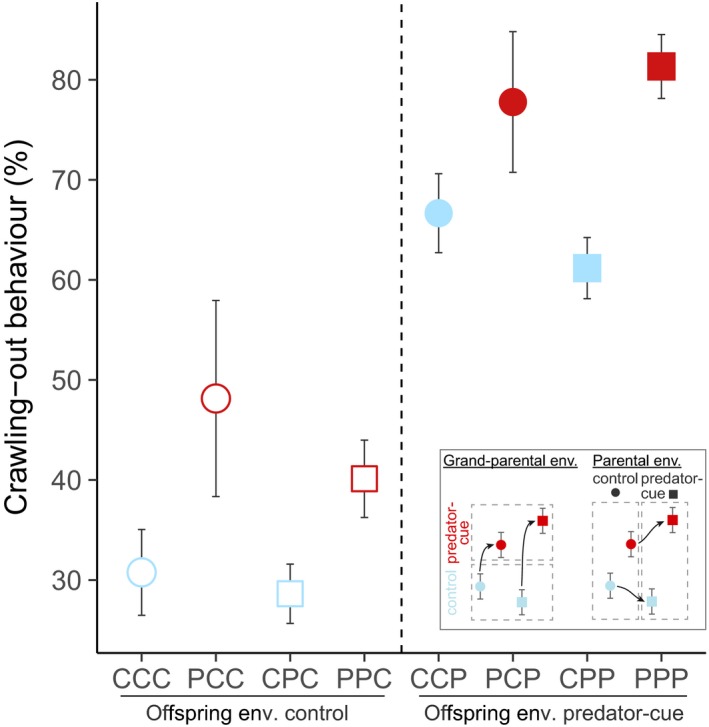
The effect of multigenerational exposure to predator cues on offspring crawling‐out behavior (proportion of snails out the water in %). The legend panel at the bottom shows which shapes to compare to identify grand‐parental or parental environmental effect. CCC, PCC, CPC, PCC, CCP, PCP, CPP, and PPP represent the eight combinations of grand‐parental (E1), parental (E2), and offspring (E3) environments with "C" for control environment and "P" for predator‐cue environment for every generation. The vertical dashed line separates the two offspring treatment groups. Blue shapes are for grand‐parental control environment and red shapes for grand‐parental predator‐cue environment. Circles are for parental control environment and squares for parental predator‐cue environment. Open shapes are for offspring control environment and closed shapes for offspring predator‐cue environment. Data are means ± *SE*

The offspring exposure to predator cues (E3) significantly increased by 105% the proportion of snails crawling‐out the water (Table 1a; Figure 3). The parental environment (E2) did not affect the proportion of crawling‐out behavior (Table 1a; Figure 3). However, grand‐parental exposure to predator cues (E1) significantly increased by 28% the proportion of crawling‐out behavior (Table 1a; Figure 3).

**Table 1:** Results of: (a) the generalized mixed model on offspring crawling‐out behavior; (b) the linear mixed model on offspring snail size (PC1); and (c) the linear mixed model on offspring shell thickness (PC2) (PC1 and PC2 are the principal components of the principal component analysis on offspring snail mass, shell thickness, and four shell size traits)

	Fixed effects	Estimate (*SE*)	*df*	Χ^2^	*p*
a. Crawling‐out	Grand‐parental env. (E1)	0.848 (0.6812)	1	8.29	**.0040**
	Parental env. (E2)	−0.090 (0.3864)	1	0.4	.5274
	Offspring env. (E3)	1.738 (0.3544)	1	114.46	**<.0001**
	E1 × E2	−0.389 (0.7696)	1	0.01	.9315
	E1 × E3	−0.168 (0.7882)	1	1.89	.1687
	E2 × E3	−0.146 (0.4242)	1	0.01	.9072
	E1 × E2 × E3	0.855 (0.8915)	1	0.91	.3395

	Random effects	Variance	*df*	Χ^2^	*p*
	Family	0.489	1	14.98	**.0001**
	Individual	0.391	1	8.26	**.0040**

	Fixed effects	Estimate (*SE*)	Numdf, Dendf	*F*	*p*
b. Snail size (PC1)	Grand‐parental env. (E1)	0.037 (0.8837)	1, 41.93	0.47	.4986
	Parental env. (E2)	−0.212 (0.4689)	1, 48.96	0.45	.4528
	Offspring env. (E3)	−0.915 (0.4717)	1, 355.06	17.40	**<.0001**
	E1 × E2	−0.183 (0.9912)	1, 56.62	0.10	.7589
	E1 × E3	−0.150 (1.0779)	1, 349.40	0.18	.6695
	E2 × E3	0.052 (0.5811)	1, 363.02	0.005	.9463
	E1 × E2 × E3	−0.075 (0.2114)	1, 358.88	0.003	.9505

	Random effect	Variance	*df*	Χ^2^	*p*
	Family	0.3572	1	4.60	**.0320**

	Fixed effects	Estimate (*SE*)	Numdf, Dendf	*F*	*p*
c. Shell thickness (PC2)	Grand‐parental env. (E1)	0.055 (0.2821)	1, 41.14	0.11	.7409
	Parental env. (E2)	−0.227 (0.1484)	1, 49.44	0.06	.8066
	Offspring env. (E3)	0.313 (0.1543)	1, 356.26	45.04	**<.0001**
	E1 × E2	0.103 (0.3155)	1, 58.24	0.48	.4923
	E1 × E3	−0.406 (0.3535)	1, 350.40	4.11	**.0433**
	E2 × E3	0.410 (0.1904)	1, 363.97	6.67	**.0102**
	E1 × E2 × E3	0.099 (0.3975)	1, 359.66	0.06	.8036

	Random effect	Variance	*df*	Χ^2^	*p*
	Family	0.026	1	1.72	.1892

Note

Bold values indicate significant *p*‐values (*p* < .05).

### 3.2 Snail mass, shell thickness, and shell size

**Figure 4:**
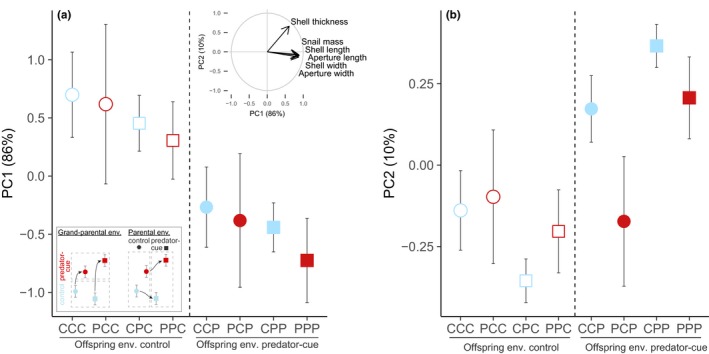
The effect of multigenerational exposure to predator cues on offspring (a) snail size (principal component PC1) and (b) shell thickness corrected for shell size (principal component PC2). The correlation circle of the principal component analysis on offspring snail mass, shell thickness, and four shell size traits is shown at the top. The legend panel at the bottom left shows which shapes to compare to identify grand‐parental or parental environmental effect. CCC, PCC, CPC, PCC, CCP, PCP, CPP, and PPP represent the eight combinations of grand‐parental (E1), parental (E2), and offspring (E3) environments with "C" for control environment and "P" for predator‐cue environment for every generation. The vertical dashed line separates the two offspring treatment groups. Blue shapes are for grand‐parental control environment and red shapes for grand‐parental predator‐cue environment. Circles are for parental control environment and squares for parental predator‐cue environment. Open shapes are for offspring control environment and closed shapes for offspring predator‐cue environment. Data are means ± *SE*

Principal component analysis revealed that 96% of the variance for snail mass, shell thickness, and four shell size traits was explained by the first and second principal components (PC1 and PC2; Figure 4). We interpreted PC1 as a proxy of snail size: PC1 was mostly driven by snail mass (18%), shell length (19%), shell width (19%), aperture length (18%), and aperture width (17%) and was slightly driven by shell thickness (9%). PC2 was driven mostly by shell thickness (91%) allowing to interpret this axis as a proxy of shell thickness corrected for snail size.

The offspring exposure to predator cues significantly reduced the snail size (PC1; Table 1b; Figure 4a) but neither the parental environment nor the grand‐parental one influenced it (Table 1b; Figure 4a).

Offspring environment interacted both with grand‐parental and parental environments to shape the shell thickness (PC2; Table 1c; Figure 4b). In the offspring control environment, the grand‐parental exposure to predator cues increased the offspring shell thickness (PC2) whereas the parental exposure to predator cues decreased it (Figure 4b). In the offspring predator‐cue environment, the grand‐parental exposure to predator cues decreased the offspring shell thickness (PC2) whereas the parental exposure to predator cues increased it (Figure 4b). Regarding the direct effect of offspring environment, offspring from predator‐cue environment had a thicker shell (PC2) than those from current control environment (Figure 4b).

## 4 DISCUSSION

We first confirm that the exposure to predator cues induces well‐known defences against crayfish predation in *P. acuta* (Auld & Relyea, 2011; Dalesman, Rundle, & Cotton, 2009; DeWitt, Robinson, & Wilson, 2000; DeWitt et al., 1999; Turner, Fetterolf, & Bernot, 1999). The offspring exposure to predator cues induced higher crawling‐out behavior, shell‐crushing resistance (thicker shell) and entry‐resistant shell (narrower shell and aperture). Moreover, offspring exposed to predator cues were lighter, suggesting a trade‐off, that is, a lower energetic investment in growth due to a potential cost to produce these defences (as shown in other gastropod species: Brönmark et al., 2012). This result stresses the fitness advantage of within‐generational plasticity which allow the production of costly defences only in case of predation (Harvell, 1990). Our key finding is that predator cues alter also offspring defences two generations later but depending on the offspring environment (within‐X transgenerational plasticity) and the defensive traits considered. Our experimental work highlights that transgenerational plasticity effects can be complex beyond the parental generation and that the offspring phenotype results from a combination of multigenerational effects.

### 4.1 Grand‐parental and parental effects on anti‐predator defences

Transgenerational plasticity is expected to evolve when the ancestral environment is a good proxy of offspring environment (Bonduriansky & Day, 2009; Dey, Proulx, & Teotónio, 2016; English, Pen, Shea, & Uller, 2015; Harvell, 1990; Leimar & McNamara, 2015; Uller, 2008), allowing a preadaptation of offspring to predation risk (Agrawal et al., 1999). In our predator‐prey system, crayfish has a long lifespan (ca. 3 years) compared to the generation time of *P. acuta* (ca. 50 days) and a relatively sedentary lifestyle (Vioque‐Fernández, Alves de Almeida, & López‐Barea, 2009). This suggests that generational cues of crayfish presence can be a good proxy of predation risk across several snail generations and thus that transgenerational plasticity could have long‐lasting effects on the antipredator defences. Consistently, in *P. acuta*, parental exposure to predator cues induces a more crush‐resistant shell and a higher escape behavior in offspring (Beaty et al., 2016; Luquet & Tariel, 2016). In this study, as expected, transgenerational plasticity went further than the parental generation: The grand‐parental environment also influenced the escape behavior and the shell thickness of offspring.

How long can persist transgenerational effects on anti‐predator responses remains an open question. To our knowledge, the study of Sentis et al. (2018) on the pea aphid (*Acyrthosiphon pisum*) is the only one to investigate predator‐induced transgenerational plasticity over many generations (>25). They found that the defensive phenotype—a high frequency of winged aphids in the population—persists for one generation after removing predators whatever the induction time is, *that is,* the previous number of successive generations experiencing the novel environment (predator presence). However, three generations are needed after removing predators for the frequency of winged phenotypes to come back to the control level, and this number of generations increases with the induction time. Together, these results suggest that multigenerational environmental effects on inducible defences are broader than just a parental effect and could persist for many generations.

### 4.2 Combination of multigenerational effects on antipredator defences

We showed that the offspring phenotype results from a combination of multigenerational effects (grand‐parents, parents and offspring), similar to theoretical and other experimental studies (Burggren, 2015; Hafer et al., 2011; Kou et al., 2011; Lock, 2012; Prizak et al., 2014; Shama & Wegner, 2014; Walsh et al., 2014). However, in our study, grand‐parental and parental effects acted independently (no significant interaction between grand‐parental and parental environmental effects): Either only one affected the offspring environment (behavior), or in interaction with the offspring environment (within‐X transgenerational plasticity) and in opposite directions (shell thickness). This results in complex offspring phenotypic patterns that do not fit with a self‐explanatory antipredator scenario with clear adaptive advantages. It would be thus interesting to assess the adaptive relevance by comparing the survival of snails from different past environmental histories exposed to lethal predation challenges. The offspring crawling‐out behavior increased with offspring and grand‐parental exposures to predator cues while the parental environment did not alter this behavior. Abnormal mortality in one lineage (lineage with grand‐parental exposure but no parental exposure) might explain why parental effects were not detected on behavior. Shell thickness was influenced by both grand‐parental and parental environments, but in opposing directions and depending on the offspring environment (grand‐parental and parental within‐X transgenerational plasticity interactions). In offspring control environment, grand‐parental exposure to predator cues increased the offspring shell thickness whereas parental exposure reduced it. The effects were opposite in the offspring predator‐cue environment.

Firstly, these results confirm that offspring reaction norms can be altered by parental environment (shell thickness; Donelson et al., 2018; Luquet & Tariel, 2016; Salinas et al., 2013) but expand for the first time the within‐X transgenerational plasticity interaction to grand‐parental environmental cues (shell thickness). Secondly, the apparent discrepancy between grand‐parental and parental effects for the crawling‐out behavior or the opposing directions found on shell thickness is not rare in empirical studies (Magiafoglou & Hoffmann, 2003; Shama & Wegner, 2014) and illustrates the complexity in determining the adaptive significance of multigenerational effects. Such discrepancies may reflect different mechanisms underlying the transfer of environmental information (Shea, Pen, & Uller, 2011). This complex opposing relationship between grand‐parental and parental environmental effects could be also theoretically beneficial by reducing the phenotypic variance which allow the population to stay closer to the target phenotype (Prizak et al., 2014). Moreover, focusing on few generations in short‐term experiments artificially focuses the interpretations of such effects while they could only be transient over longer timescales in a population dynamic framework. For example, Sentis et al. (2018), after removing predators, observed that the frequency of winged aphids remained high for one generation before dropping abruptly below the control levels (grand‐parental effect), and then converging with the winged aphid frequencies of the control lines (great‐grand‐parental effect). Consequently, in focusing on only three consecutive generations as in our study, these results could be interpreted as a negative grand‐parental effect (decrease of winged aphid frequency) opposing to a positive parental effect (increase of winged individual frequency) on the offspring phenotype. These findings highlight the need to develop empirical studies on longer timescales and controlling for the combination of multigenerational effects.

### 4.3 Trait‐dependence of transgenerational plasticity

Our results show that the pattern of transgenerational plasticity depends on the traits (escape behavior, shell thickness and shell size). Behavioral traits, which are often labile and exhibiting reversible within‐generational plasticity within developmental or adult stages, are predicted to be influenced by current environment rather than by past environmental experience (Dingemanse & Wolf, 2013; Piersma & Drent, 2003). Behavioral within‐generational plasticity in response to current environmental cues should rapidly by‐pass the behavioral transgenerational plasticity (Beaman, White, & Seebacher, 2016). By contrast, the traits that are more constrained during the development and exhibiting irreversible variations, as morphological traits, are predicted to be relatively more influenced by past environments (Kuijper & Hoyle, 2015). Transgenerational plasticity on morphological traits could irreversibly engage the offspring on developmental trajectories and could not be compensated by within‐generational plasticity. In *P. acuta*, crawling‐out behavior is indeed very flexible and reversible at a time scale of hours while a thicker shell and a narrower shell shape are irreversible changes in the developmental trajectory (DeWitt et al., 1999; Relyea, 2003). Surprisingly in our study, the escape behavior of offspring is influenced by the grand‐parental environment while shell size was not influenced by parental or grand‐parental environments. This highlights that transgenerational effects on morphological traits may have a short persistence over generations while behavioral transgenerational plasticity may be much more prevalent than currently realized. Parental transgenerational plasticity on behavioral traits has been sometimes observed (e.g., Bestion, Teyssier, Aubret, Clobert, & Cote, 2014; Donelan & Trussell, 2015; Giesing, Suski, Warner, & Bell, 2011; Storm & Lima, 2010) and few times with long‐lasting effects over generations (Dias & Ressler, 2014; Remy, 2010).

## CONFLICT OF INTEREST

The authors of this preprint declare that they have no conflict of interest with the content of this article.

## AUTHOR CONTRIBUTIONS

All authors contributed to study design, data collection, analysis, and interpretation. All authors drafted, revised, and approved the article for publication.

### Open Research Badges

This article has been awarded Open Materials, Open Data Badges. All materials and data are publicly accessible via the Open Science Framework at http://dx.doi.org/10.5281/zenodo.3573840

## Data Availability

Data and R script are available from the Zenodo repository (http://dx.doi.org/10.5281/zenodo.3573840).
